# Pharmacological effects of the Cassia Seed on atherosclerosis: A meta-analysis based on network pharmacology

**DOI:** 10.1097/MD.0000000000030411

**Published:** 2022-09-09

**Authors:** Sen Zhang, Sijing Rao, Mei Wen Yang, Ya-Ting Huang, Fen-Fang Hong, Shu-Long Yang

**Affiliations:** a Department of Physiology, College of Medicine, Nanchang University, Nanchang, China; b Department of Surgery, Fuzhou Medical College, Nanchang University, Jiangxi, Fuzhou, China; c Experimental Center of Pathogen Biology, Nanchang University, Nanchang, China; d Key Research Laboratory of Chronic Diseases, Fuzhou Medical College, Nanchang University, Fuzhou, China; e Department of Physiology, Fuzhou Medical College, Nanchang University, Jiangxi, Fuzhou, China.

**Keywords:** atherosclerosis, bioinformatics, Cassia Seed, network pharmacology

## Abstract

The aim of this study was to shed light on the active ingredients and potential targets of Cassia Seed about anti-atherosclerosis based on network pharmacology.

**Methods:**

The active ingredients and potential targets of Cassia Seed were obtained from traditional Chinese medicine systems pharmacology database and analysis platform (TCMSP) and SwissTargetPrediction database. Then, atherosclerosis-related targets were screened via GeneCards, online mendelian inheritance in man, therapeutic target database and DrugBank database. The common targets and protein–protein interaction (PPI) network was later identified and built. Furthermore, we used the database for annotation, visualization and integrated discovery (DAVID) database server to accomplish the enrichment analysis. The compounds-targets-pathways network was ultimately constructed by Cytoscape.

**Results:**

A total of 14 active ingredients and 475 related targets were sifted from Cassia Seed. Among 574 potential atherosclerotic targets, there were 99 targets overlapped with those of Cassia Seed. Topological analysis with Cytoscape revealed that proto-oncogene tyrosine-protein kinase proto-oncogene tyrosine-protein kinase Src, transcription factor AP-1 (JUN), mitogen-activated protein kinase 8 (MAPK8), mitogen-activated protein kinase 14 (MAPK14) and catenin beta-1 were considered as the hub gene. Gene ontology (GO) and Kyoto encyclopedia of genes and genomes (KEGG) enrichment analysis suggested that the Cassia Seed had the potential to influence varieties of biological processes and pathways, including positive regulation of smooth muscle cell proliferation, inflammatory response, tumor necrosis factor (TNF) signaling pathway, vascular endothelial growth factor (VEGF) signaling pathway and arachidonic acid (ARA) metabolism.

**Conclusion:**

Taken together, our findings support that anti-atherosclerosis effects of Cassia Seed are characterized by multi-component, multi-target and multi-path mechanism of action.

## 1. Introduction

Atherosclerosis (AS), a type of cardiovascular disease (CVD), is a fatal pathological variation to human health and a leading cause of morbidity and mortality worldwide.^[1]^ The underlying pathogenesis of AS involves a series of pathological processes including lipid metabolism disorders, inflammation, oxidative stress, and foam cell formation.^[2,3]^ Disturbingly, 2.4 million individuals died of AS in China in 2016, accounting for 61% of CVD deaths in the same year.^[4]^ Nevertheless, the incidence and mortality of AS are still rising by now, even with the improvement of lifestyle and the use of lipid-lowering strategies.

Cassia Seed, also known as semen cassiae, is the dried mature seeds of Cassia obtusifolia L. or Cassia tora L. It is recorded in Shennong’s herbal classics that Cassia Seed has the effect of improving eyesight, relaxing the bowels, and is mainly used for dry eyes, headache, fainting vertigo and constipation.^[5,6]^ A number of modern pharmacological studies have elucidated that Cassia Seed relieves the increase of lipid level and blood pressure, and play a role in anti-inflammation, antioxidation and antibacteria.^[7–9]^

In traditional Chinese medicine, Cassia Seed has long been clinically used for diabetes and hyperlipidemia conditions. However, the molecular mechanisms underlying the anti-atherosclerosis effects in Cassia Seed have not been clearly defined, so further investigations should be carried out without delay. Therefore, this study provides the prediction of interactions between AS related targets and the active ingredients of Cassia Seed, which may highlight potential targets for further molecular studies, uncover the beneficial components of Cassia Seed and lead to the development of an effective treatment for AS.

## 2. Methods

### 2.1. Data preparation

#### 2.1.1. Herbal compounds and compound targets in Cassia Seed.

Traditional Chinese medicine systems pharmacology database and analysis platform (TCMSP, https://tcmspw.com/tcmsp.php) were used to obtain the components of Cassia Seed. As TCMSP suggests, the active ingredients with oral bioavailability ≥30% and drug-likeness ≥0.18 were selected for subsequent analysis. The potential targets of Cassia Seed were screened from the following databases: TCMSP, PubChem (https://pubchem.ncbi.nlm.nih.gov/), SwissTargetPrediction (http://www.swisstargetprediction.ch/), and PharmMapper (http://www.lilab-ecust.cn/pharm mapper/). The resulting target protein names were converted into a unified format according to the Unified Protein Database (https://www.uniprot.org/).

#### 2.1.2. Atherosclerosis targets.

Details on human genes associated with AS were collected from the following sources: GeneCards (http://genecards.org), Online Mendelian Inheritance in Man (http://www.omim.org), Therapeutic Target Database (http://bidd.nus.edu.sg/group/cjttd) and DrugBank databases (http://www.drugbank.ca). Here, “atherosclerosis” was used as the keyword to collect known targets from these 4 databases for the species “Homo sapiens.”

### 2.2. PPI network

The common targets of Cassia Seed and AS were singled out by *R* × 4.0.2 software. These common targets were used to construct a protein–protein interaction (PPI) network on the Search Tool for the Retrieval of Interacting Genes/Proteins database (https://string-db.org/) with the “Homo sapiens” setting. In this study, we set the minimum value of the confidence at “highest confidence (>0.9)” and removed the disconnected proteins from the network.

### 2.3. Network visualization and topological analysis

The PPI information were exported in TSV format, then visualized by Cytoscape 3.7.2 software. The Cytoscape tool “Network Analyzer” was applied to visualize the topological properties, and the hub genes in the PPI network were screened out based on these properties. Other significant topological parameters were calculated by the CytoHubba App in Cytoscape. The cluster analysis algorithm MCODE was performed to analysis clustering modules in the PPI network.

### 2.4. Enrichment analysis

Both gene ontology (GO) and Kyoto Encyclopedia of Genes and Genomes (KEGG) enrichment analysis were conducted based on the Database for Annotation, Visualization and Integrated Discovery (DAVID, https://david.ncifcrf.gov/). Subsequently, the top 10 results of each enriched term or pathway were visualized with *R* × 4.0.2 software.

### 2.5. Compounds-targets-pathways network construction

We prepared the integrated excel file of the Cassia Seed active ingredients, common targets and the first 20 KEGG pathways that the Cytoscape software need. After running the corresponding method, the network was obtained. Finally, set “Style” according to the degree value to distinguish the importance of each active ingredient, target, and pathway.

### 2.6. Statistical analyses

All statistical data analyses were performed based on the online databases, Cytoscape 3.7.2 and *R* × 4.0.2 software algorithm.

### 2.7. Ethical review

Ethical approval was not necessary because this study did not involve any animal, human, or cell experiments.

## 3. Result

### 3.1. Screening of active compounds and targets

A total of 14 effective active compounds of Cassia Seed were collected in the TCMSP database (Table 1) (Table S1, Supplemental Digital Content 1, http://links.lww.com/MD/H198). After striking out redundant targets, there were 475 targets corresponding to the active compounds (Table S2, Supplemental Digital Content 2, http://links.lww.com/MD/H199).

**Table 1:** The active compounds of Cassia Seed.

Mol ID	Compound name	OB (%)	DL	Structure
MOL000471	aloe-emodin	83.38	0.24	
MOL006475	Obtusin	81.43	0.4	
MOL006482	9,10-dihydroxy-7-methoxy-3-methylene-4H-benzo[g]isochromen-1-one	63.25	0.24	
MOL006489	Quinizarin	47.34	0.19	
MOL002268	rhein	47.07	0.28	
MOL002281	Toralactone	46.46	0.24	
MOL006466	Rubrofusarin	45.55	0.24	
MOL000449	Stigmasterol	43.83	0.76	
MOL006481	gluco-obtusifolin	42.41	0.81	
MOL006465	Rubrofusarin-6-beta-gentiobioside	40.12	0.67	
MOL000953	CLR	37.87	0.68	
MOL005043	campest-5-en-3beta-ol	37.58	0.71	
MOL006472	Aurantio-obtusin	31.55	0.37	
MOL006486	obtusin	31.24	0.4	No structure found

DL = drug-likeness, OB = oral bioavailability.

### 3.2. Candidate genes associated with AS and common targets

**Figure 1.**
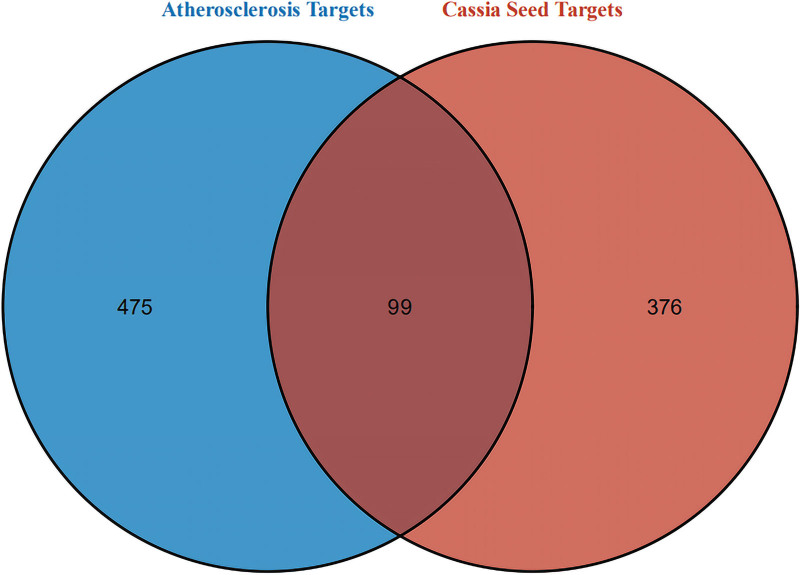
Intersection targets of AS and Cassia Seed. The blue circle represents the atherosclerosis target, and the red circle represents the cassia seed targets. AS = atherosclerosis.

The GeneCards, online mendelian inheritance in man, therapeutic target database and DrugBank databases were used to identify reviewed therapeutic targets in AS. As a result, 574 AS-related targets were acquired (Table S2, Supplemental Digital Content 2, http://links.lww.com/MD/H199). With the help of *R* × 4.0.2 software, 99 target genes were identified affected by AS and regulated by Cassia Seed (Fig. 1) (Table S2, Supplemental Digital Content 2, http://links.lww.com/MD/H199).

### 3.3. Construction and topological analysis of PPI network

**Figure 2.**
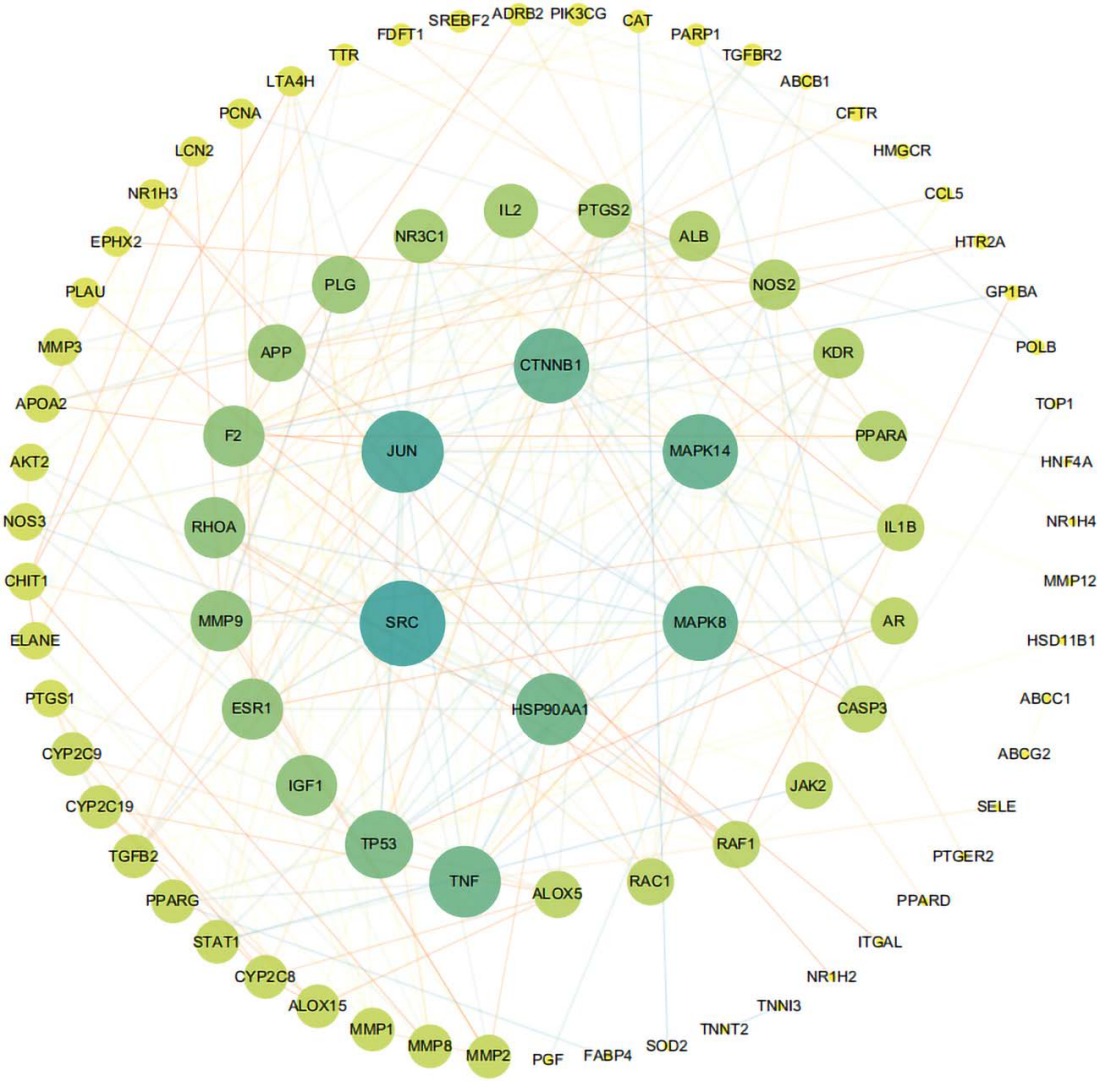
Protein–protein interaction network analysis of Cassia Seed for the treatment of AS. The larger the shape, the higher the degree of the target. AS = atherosclerosis.

**Figure 3.**
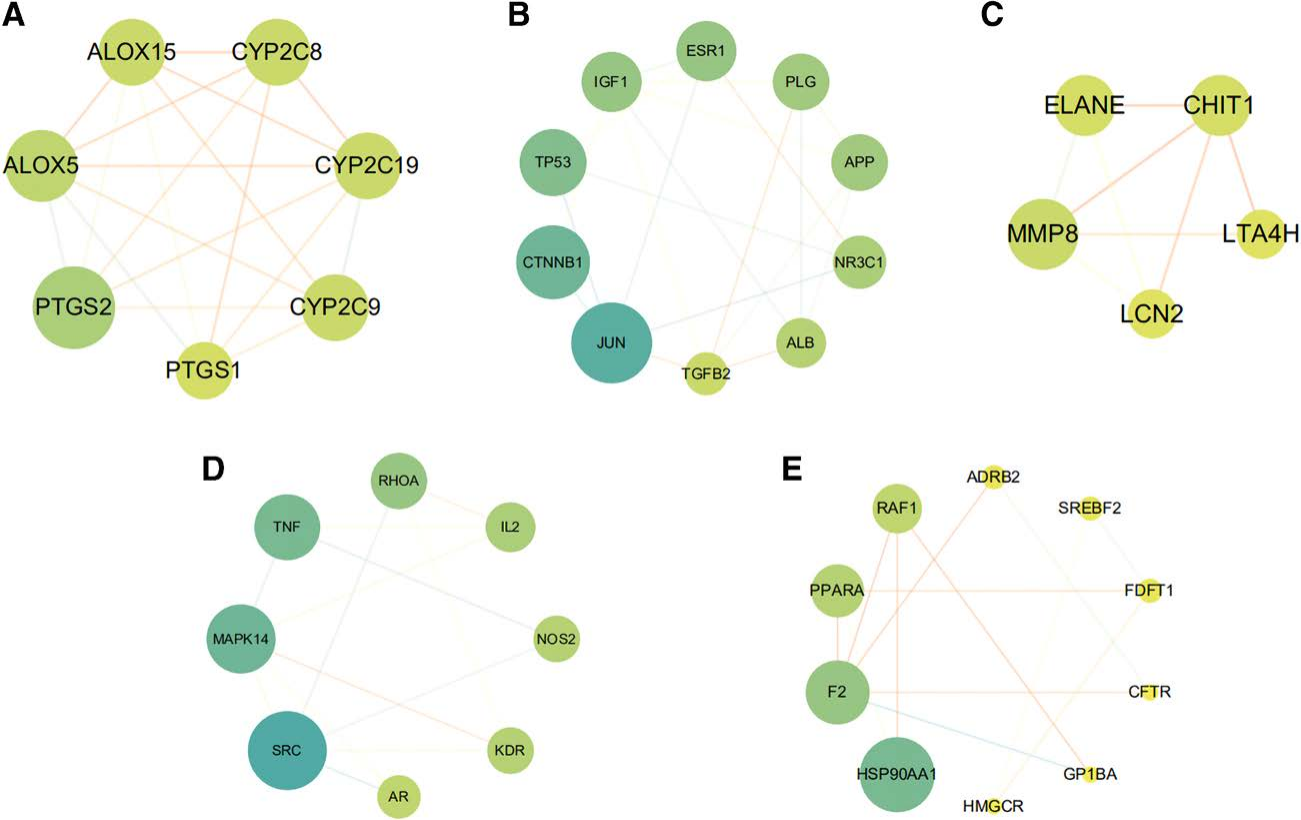
MCODE analysis of PPI network. (A) Cluster 1, composed of 7 nodes and 19 edges (score = 6.333). (B) Cluster 2, composed of 10 nodes and 20 edges (score = 4.444). (C) Cluster 3, composed of 5 nodes and 8 edges (score = 4.000). (D) Cluster 4, composed of 8 nodes and 13 edges (score = 3.714). (E) Cluster 5, composed of 10 nodes and 13 edges (score = 2.889). PPI = protein–protein interaction.

A PPI network of those 99 common targets were then established via search tool for the retrieval of interacting genes/proteins database. Next, the Cytoscape software was used to visualize the PPI network (Fig. 2). Topological analysis results manifested that there were 84 nodes and 239 edges in total, and the average value of degree, betweenness centrality and closeness centrality is 8, 0.032 and 0.351 respectively. As showed in Table 2, proto-oncogene tyrosine-protein kinase Src, transcription factor AP-1 (JUN), mitogen-activated protein kinase 8 (MAPK8), mitogen-activated protein kinase 14 (MAPK14), catenin beta-1 and HSP90AA1 were identified as hub genes. The topological parameters (MCC, DMNC, MNC, Degree, EPC, BottleNeck, EcCentricity, Closeness, Radiality, Betweenness, Stress, Clustering Coefficient) were calculated by CytoHubba (Table S3, Supplemental Digital Content 3, http://links.lww.com/MD/H200). Based on MCODE algorithm, the targets were classified into 5 separated clusters (Fig. 3) (Table S4, Supplemental Digital Content 4, http://links.lww.com/MD/H201).

**Table 2:** Top 20 targets ranked by degree value.

Name	Degree	Betweenness centrality	Closeness centrality
SRC	18	0.164	0.479
JUN	17	0.130	0.459
MAPK8	15	0.064	0.446
MAPK14	15	0.036	0.429
CTNNB1	15	0.097	0.422
HSP90AA1	14	0.041	0.420
TNF	14	0.128	0.449
TP53	13	0.092	0.411
F2	11	0.115	0.391
RHOA	11	0.027	0.389
MMP9	11	0.110	0.411
ESR1	11	0.040	0.418
IGF1	11	0.056	0.407
PLG	10	0.054	0.362
APP	10	0.055	0.387
NR3C1	9	0.003	0.389
PTGS2	9	0.114	0.369
IL2	9	0.007	0.387
KDR	8	0.027	0.369
NOS2	8	0.102	0.407

CTNNB1 = catenin beta-1, HSP90AA1 = Heat shock protein HSP 90-alpha, IGF1 = insulin-like growth factor I, JUN = transcription factor AP-1, MAPK8 = mitogen-activated protein kinase 8, MAPK14 = mitogen-activated protein kinase 14, MMP9 = matrix metalloproteinase-9, RHOA = transforming protein RhoA, SRC = proto-oncogene tyrosine-protein kinase Src, TNF = tumor necrosis factor.

### 3.4. GO enrichment analyses and KEGG pathway analyses

GO enrichment analysis of the common targets was implemented using DAVID database. As suggested from the results that 481 enriched GO terms were obtained, including 340 biological processes, 43 cellular components and 98 molecular functions (Table S5, Supplemental Digital Content 5, http://links.lww.com/MD/H202). To identify pathways that are related to the effects of Cassia Seed in AS treatment, the KEGG pathway enrichment analysis was performed. From DAVID database, we found 104 pathways were enriched (Table S5, Supplemental Digital Content 5, http://links.lww.com/MD/H202).

#### 3.4.1. GO enrichment analyses.

**Figure 4.**
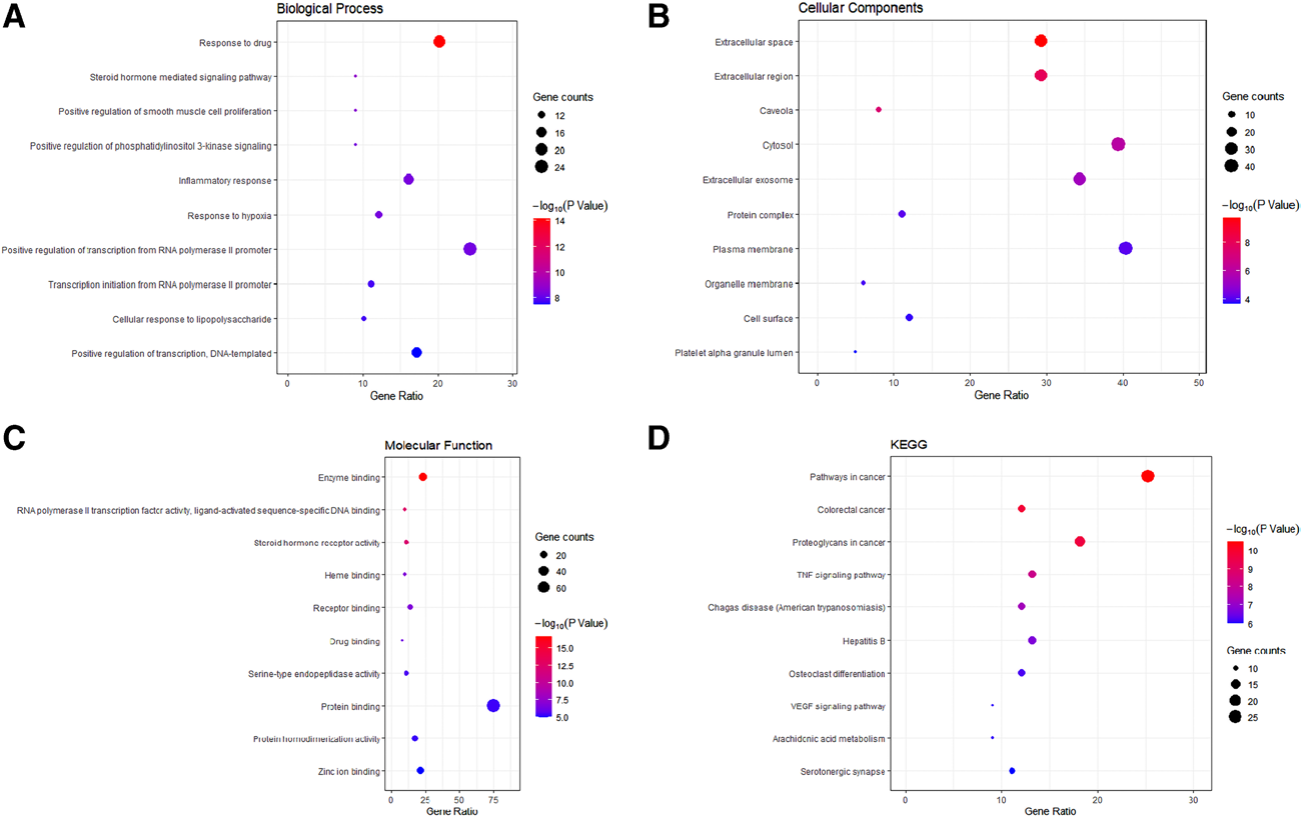
GO and KEGG pathway enrichment analyses. (a) The top 10 significant enriched terms in biological process (BP). (b) The top 10 significant enriched terms in cellular component (CC). (c) The top 10 significant enriched terms in molecular function (MF). (d) The top 10 significant enriched pathways in Kyoto Encyclopedia of Genes and Genomes (KEGG). GO = gene ontology.

The biological processes results (Fig. 4A) suggested that these targets mainly respond to drug reaction (GO:0042493), steroid hormone mediated signaling pathway (GO:0043401), positive regulation of smooth muscle cell proliferation (GO:0048661), positive regulation of phosphatidylinositol 3-kinase signaling (GO:0014068) and inflammatory response (GO:0006954). Cellular components results (Fig. 4B) included the extracellular space (GO:0005615), extracellular region (GO:0005576), caveola (GO:0005901), cytosol (GO:0005829) and extracellular exosome (GO:0070062). For molecular functions (Fig. 4C), the targets were mostly involved in enzyme binding (GO:0019899), RNA polymerase II transcription factor activity, ligand-activated sequence-specific DNA binding (GO:0004879), steroid hormone receptor activity (GO:0003707), heme binding (GO:0020037) and receptor binding (GO:0005102).

#### 3.4.2. KEGG pathway enrichment analyses.

According to the analysis of KEGG pathways, these common targets were enriched in multiple pathways known to participate in the progression of AS, such as tumor necrosis factor (TNF) signaling pathway (hsa04668), vascular endothelial growth factor (VEGF) signaling pathway (hsa04370) and arachidonic acid (ARA) metabolism (hsa00590) (Fig. 4D and Table 3).

**Table 3:** Results of KEGG pathway enrichment analysis.

Term	*P* value	Genes
Pathways in cancer	10.465	PTGER2, PTGS2, PIK3CG, MAPK8, CASP3, AKT2, RAC1, TGFB2, JUN, HSP90AA1, NOS2, MMP1, STAT1, MMP2, IGF1, MMP9, RHOA, PGF, TGFBR2, AR, CTNNB1, PPARG, RAF1, TP53, PPARD
Colorectal cancer	9.736	TGFB2, JUN, MAPK8, AKT2, CASP3, CTNNB1, RAC1, RAF1, TP53, RHOA, PIK3CG, TGFBR2
Proteoglycans in cancer	9.581	TGFB2, SRC, MMP2, IGF1, MAPK14, ESR1, TNF, MMP9, RHOA, PIK3CG, PLAU, CASP3, AKT2, KDR, CTNNB1, RAC1, RAF1, TP53
TNF signaling pathway	8.201	JUN, MMP3, PTGS2, MAPK14, SELE, TNF, MMP9, PIK3CG, MAPK8, IL1B, CASP3, AKT2, CCL5
Chagas disease (American trypanosomiasis)	7.278	TGFB2, JUN, MAPK8, NOS2, AKT2, CCL5, IL1B, MAPK14, TNF, IL2, PIK3CG, TGFBR2
Hepatitis B	6.708	TGFB2, JUN, PCNA, STAT1, SRC, TNF, MMP9, PIK3CG, MAPK8, CASP3, AKT2, RAF1, TP53
Osteoclast differentiation	6.244	TGFB2, JUN, MAPK8, STAT1, AKT2, IL1B, PPARG, RAC1, MAPK14, TNF, PIK3CG, TGFBR2
VEGF signaling pathway	6.132	SRC, NOS3, AKT2, KDR, RAC1, RAF1, MAPK14, PTGS2, PIK3CG
Arachidonic acid metabolism	6.132	CYP2C9, CYP2C8, ALOX5, EPHX2, ALOX15, LTA4H, CYP2C19, PTGS2, PTGS1
Serotonergic synapse	5.995	CYP2C9, APP, CYP2C8, ALOX5, CASP3, ALOX15, HTR2A, CYP2C19, RAF1, PTGS2, PTGS1

CTNNB1 = catenin beta-1, HSP90AA1 = Heat shock protein HSP 90-alpha, IGF1 = insulin-like growth factor I, JUN = transcription factor AP-1, KEGG = Kyoto Encyclopedia of Genes and Genomes, MAPK8 = mitogen-activated protein kinase 8, RHOA = transforming protein RhoA.

### 3.5. Compounds-targets-pathway network construction

**Figure 5.**
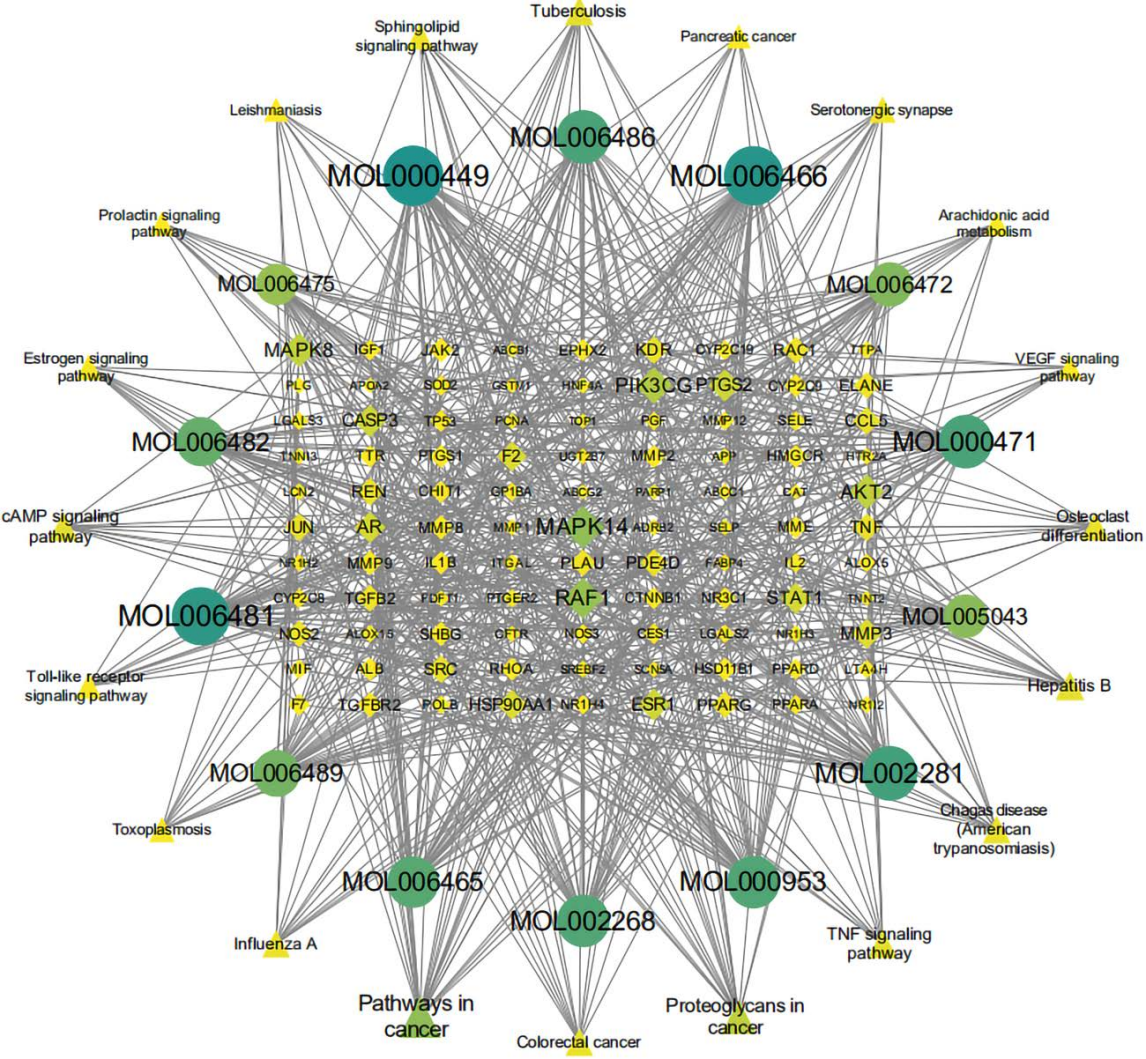
The compounds-targets-pathways network. The diamond represents the common targets. The circle target represents the active ingredients. The triangle represents the KEGG pathways. KEGG = Kyoto encyclopedia of genes and genomes.

To comprehensively reveal the molecular mechanisms underlying the effects of Cassia Seed against AS further, a compounds-targets-pathways network was built. Based on this network, 133 nodes (14 compounds, 99 common targets and 20 pathways) and 704 edges were connected. As showed in Figure 5, the triangle, circle and diamond nodes represent pathways, active compounds and common targets, respectively. The larger shape and label meant stronger interactions.

## 4. Discussion

AS is the pathological basis of CVD, such as coronary heart disease, cerebral infarction and peripheral vascular disease.^[10]^ AS has long been recognized as a complex pathological process involving lipid metabolism disorder, inflammation, oxidative stress and foam cell formation, and characterized by the accumulation of lipids and calcium in the arterial intima.^[11]^ Modern pharmacological studies have shown that the main components of Cassia Seed, anthraquinones and naphthapyrones, significantly reduce the level of blood lipids and have the effects of anti-inflammation and antioxidation.^[9,12]^ Previous researches have also reported that Cassia Seed plays a positive role in hyperlipidemia.^[13]^ However, the relationship between Cassia Seed and AS has not been thoroughly studied and requires further researches.

Via systems pharmacologic analysis, 14 active ingredients of Cassia Seed with oral bioavailability ≥30% and drug-likeness ≥0.18 were selected from TCMSP database. These active ingredients were classified into the following 2 categories: anthraquinone: aloe-emodin, Obtusin, rhein, gluco-obtusin and aurantio-obtusin; naphthapyrone: rubrofusarin, toralactone and rubrofusarin-6-beta-gentiobioside. Anthraquinone has long been identified as the vital active compounds of Cassia Seed. Existing in vitro and in vivo studies have indicated that quercetin is capable of inhibiting inflammatory response and reducing the level of blood lipids.^[14,15]^

Based on the PPI network, 99 target genes were selected, which are both affected by AS and regulated by Cassia Seed. The degree value of JUN, MAPK8, MAPK14, HAP90AA1, TNF, transforming protein RhoA, matrix metalloproteinase-9, insulin-like growth factor I and nitric oxide synthase was significantly elevated and closely related to the progression and treatment of AS. V-jun avian sarcoma virus 17 oncogene homolog (JUN) belong to the family of activin-1 that act as the stimulater of proliferation, differentiation and migration of vascular smooth muscle cells.^[16]^ As such, JUN have attracted much interest as potential drug targets for alleviation of AS. This is mostly due to their potential as anti-inflammatory remedy through modulation of angiotensin II and nuclear factor-KB (NF-KB).^[17,18]^ MAPK8 is a Serine/threonine protein kinase which belongs to recombinant mitogen activated protein kinase (MAPK) family. MAPK8 is activated by the presence of malfolded proteins in the endoplasmic reticulum and upregulated inflammatory cytokines and reactive oxygen species, then it stimulates the expression of a series of downstream genes after activating the JUN through phosphorylation.^[19,20]^ Thus, MAPK8 plays an important role in regulating inflammation and oxidative stress. As for MAPK14, previous studies confirmed that MAPK14 is phosphorylated to pp38, thereby participating in the transcriptional regulation of inflammatory cells.^[21]^ There are 2 TNF isoforms, alpha and beta, which are function as the key mediators of inflammatory response owing to the capacity of activating the inflammatory mediators.^[22]^ TNF is also involved in damaging the integrity of the vascular endothelial structure and function, the proliferation of vascular smooth muscle cells, and the imbalance of coagulation and anticoagulation systems in the initial stage of AS.^[23]^ Heat shock protein HSP 90-alpha (HAP90AA1), a cytoplasmic protein, is the most widely studied protein in the heat shock protein family. Regarding the impact of HSP90AA1 on AS, previous research confirmed that HSP90AA1 protein is highly expressed in foam cells induced by ox-LDL.^[24]^ On the other hand, inhibition of HAP90AA1 reduces the expression of pro-inflammatory mediator by inhibiting the activation of NF-KB pathway.^[25]^ Other genes with higher degree value, such as transforming protein RhoA, matrix metalloproteinase-9, insulin-like growth factor I and nitric oxide synthase, are associated with changes in improving oxidative stress, vascular endothelial function damage, inflammatory cell infiltration, vascular smooth muscle cell proliferation and angiogenesis.^[26–31]^ In a word, the topological analysis shows that Cassia Seed exert beneficial actions on AS, possibly by reducing inflammation, suppressing oxidative stress, improving vascular endothelial function, promoting angiogenesis and so on.

Consistent with PPI results, the GO function enrichment revealed that Cassia Seed may has a curative effect in AS by regulating smooth muscle cell proliferation and inflammation. As demonstrated from the KEGG pathway analysis, Cassia Seed produces therapeutic effects on AS by regulating VEGF, TNF and ARA metabolism signaling pathways.

Endothelial cell dysfunction is one of the pathophysiological mechanisms of AS, leading to vascular contraction, platelet aggregation, oxidative stress, thrombosis and inflammation. VEGF is the primary signaling that is necessary for mediating vascular endothelial growth and plays a key role on the regulation of vascular endothelial cell proliferation, migration and activation.^[32]^ It has been reported that VEGF signaling pathway has strong specificity and high efficiency in promoting angiogenesis.^[33]^ It is well known that hypoxia has been the main promoter of angiogenesis. Experimental studies have suggested that hypoxic conditions often accompanied with an increase in the expression of VEGF in the plaques.^[34]^ TNF pathway has shown biological functions on anti-viral, immunomodulation and cell apoptosis by activating cysteine proteinase, JNK and NF-KB.^[35]^ NF-KB is usually combined with inhibitor of NF-KB in cells and exists in a non-active state.^[36]^ After being stimulated by TNF, inhibitor of NF-KB is degraded which in turn leading to the activation of NF-KB. Subsequently, many pro-inflammatory cytokines and chemokines will be released and the inflammatory response will be triggerd.^[37]^ ARA is an omega-6 polyunsaturated fatty acid and is considered the most widely distributed polyunsaturated fatty acid with crucial biological functions in the human body.^[38]^ When cells are irritated, ARA are decomposed into free form through the activation of phospholipase A2 (PLA2) and then released into the cell sap. With the assistance of metabolic enzymes, these ARA produce abounded metabolites, including prostaglandins, leukotrienes and epoxyeicosatrienoic acids. Eventually, these metabolites facilitate the occurrence and development of AS via multiple mechanisms such as platelet aggregation, vascular contraction, plaque vulnerability, foam cell formation, smooth muscle cell proliferation and migration.^[39]^

## 5. Conclusions

In summary, we conducted a comprehensive network pharmacology to demonstrate the key bioactive components and core potential targets of Cassia Seed against AS. We found that Cassia Seed could alleviate inflammatory response, reduce oxidative stress, promote angiogenesis, and enhance plaque stability in the treatment of AS. The core potential targets are proto-oncogene tyrosine-protein kinase Src, JUN, MAPK8, MAPK14, catenin beta-1 and HSP90AA1. The most representative pathways are VEGF signaling pathway, TNF signaling pathway and ARA metabolism. The results presented in this study provide bioinformatics data for the anti-atherosclerosis mechanism of Cassia Seed, promote related drug research and inspire new ideas for future anti-atherosclerosis therapy.

## Author contributions

**Conceptualization:** Sen Zhang.

**Data curation:** Sijing Rao, Mei Wen Yang, Ya-Ting Huang.

**Formal analysis:** Sen Zhang, Sijing Rao, Mei Wen Yang, Shu-Long Yang.

**Funding acquisition:** Fen-Fang Hong, Shu-Long Yang.

**Investigation:** Sen Zhang.

**Methodology:** Sen Zhang.

**Project administration:** Shu-Long Yang.

**Resources:** Fen-Fang Hong, Shu-Long Yang.

**Software:** Sen Zhang.

**Supervision:** Sijing Rao, Fen-Fang Hong, Shu-Long Yang.

**Validation:** Fen-Fang Hong, Shu-Long Yang.

**Writing – original draft:** Sen Zhang, Ya-Ting Huang.

**Writing – review & editing:** Sen Zhang, Sijing Rao, Mei Wen Yang, Ya-Ting Huang.

## Supplementary Material


